# The Association of Cardiometabolic Disease with Psychological Factors in Colombian People during the COVID-19 Pandemic: A Cross-Sectional Study

**DOI:** 10.3390/jcm10214959

**Published:** 2021-10-26

**Authors:** Patricia Alexandra García-Garro, Agustín Aibar-Almazán, Yulieth Rivas-Campo, Gloria Cecilia Vega-Ávila, Diego Fernando Afanador-Restrepo, Antonio Martínez-Amat, María Isabel Afanador-Rodríguez, Yolanda Castellote-Caballero, Fidel Hita-Contreras

**Affiliations:** 1 GIP Pedagogy Research Group, Faculty of Distance and Virtual Education, Antonio José Camacho University Institution, Santiago de Cali 760016, Colombia; palexandragarcia@admon.uniajc.edu.co (P.A.G.-G.); yulieth.rivas@correounivalle.edu.co (Y.R.-C.); gcvega@profesores.uniajc.edu.co (G.C.V.-Á.); afanador807@gmail.com (D.F.A.-R.); maria.isabel.afanador@gmail.com (M.I.A.-R.); 2 Department of Health Sciences, Faculty of Health Sciences, University of Jaén, 23071 Jaén, Spain; amamat@ujaen.es (A.M.-A.); mycastel@ujaen.es (Y.C.-C.); fhita@ujaen.es (F.H.-C.)

**Keywords:** cardiometabolic disease, COVID-19, sleep quality, depression, quality of life

## Abstract

During the COVID-19 pandemic, psychological disorders have been documented in the population, and their exacerbation in vulnerable populations such as those with Cardiometabolic Diseases (CD) might challenge health systems. This study determined psychological factors associated with CD in Colombian adults during the COVID-19 pandemic. For this purpose, 284 persons were evaluated, 142 without CD and 142 with CD. Sociodemographic data were collected, and the International Physical Activity Questionnaire (IPAQ), the SF-12v2, the Pittsburgh Sleep Quality Index (PSQI) and the Zung Self-Rating Depression Scale (ZSDS), which were integrated into an online form, were used. Through a simple and multiple logistic regression model, it was shown that CD was associated with low sleeping quality (LSQ) (OR = 3.51) and with depressive symptoms (DS) (OR = 1.98). In addition, in the group with CD, the presence of DS was related to BMI (OR = 2.45), and LSQ was related to living with persons at risk for COVID-19 (OR = 3.64) and BMI (OR = 5.88). In conclusion, this study showed that people with CD have a higher chance of presenting DS and LSQ. Furthermore, living with people at risk for COVID-19 was related to the presence of LSQ.

## 1. Introduction

The disease caused by severe acute respiratory syndrome coronavirus 2 (SARS-CoV-2) is still considered a public health emergency of international importance [1]. At the beginning of 2021 in the region of the Americas, the appearance of three new variants of the virus (VOC 202012/01; 501Y.V2 and P.1) with a high potential for transmissibility and risk of mortality intensified the concern of the organisms regarding the impact of this infection on health [2]. Latin American public health problems include social factors such as poverty, unemployment and low government budgets for dealing with pandemic situations [3]. Moreover, the fear of contagion and measures adopted, such as social distancing, brought with them an imminent psychological impact [4].

Current studies suggest that the COVID-19 pandemic is associated with a psychiatric epidemic, which requires more attention from the health community [5]. Different mental health problems were analyzed by recent research during the pandemic in the general population, [6] as well as in specific population groups; the elderly, [7] health care workers and [8] children and adolescents [9,10]. Other research included vulnerable groups such as people with chronic non-infectious diseases [11]. Despite this, research focused on this last population group continues to be rare, especially in Colombia.

Among the non-infectious chronic diseases, cardiometabolic diseases (CD) stand out, grouping several conditions such as obesity, diabetes and cardiovascular disease due to the presence of common risk factors [12]. CD have a high prevalence worldwide, especially in developing countries, such as Colombia [13]; according to data published between 2015 and 2019, in Colombians older than 18 years, the values reported were 24% for arterial hypertension [14], 18.7% for obesity [15] and 8.1–8.9% for type 2 diabetes mellitus [16].

These pathologies, which have a negative impact on the quality of life of those who suffer from them [17], and are often manifested in conjunction with psychological effects that are detrimental to mental health, such as depression/anxiety [18] and sleep disorders [19,20]. In general, the prevalence of depression (to varying degrees) in people with CD was estimated at 28% for type 2 diabetes mellitus [21] and 13–26.9% for high blood pressure [22,23,24,25] worldwide. It should be noted that there are variations between the prevalence reports of each country, as well as in the way in which depressive symptoms are reported [26], and heterogeneity of the diagnostic tools used in the different studies [25].

In particular, depression seems to play a key role in the poor prognosis of CD [27,28] since it is counted as a modifiable risk factor associated with poor adherence to treatment for high blood pressure [29,30] and for type 2 diabetes mellitus [31], which results in the worsening of these clinical pictures. This situation has become a topic of global interest, to the point that the World Health Organization (WHO) recommends implementing joint care for depression in people with non-infectious chronic diseases [32]. On the other hand, positive associations between obesity, high blood pressure and hyperglycemia with a longer duration of sleep were described in the Arab population [33]. In people with type 1 and 2 diabetes, both the amount of sleep and the quality of sleep were found to be essential for glycemic control and metabolic function [34,35], and some evidence suggests that lack of sleep would play an important role in the development of type 2 diabetes mellitus, mediated by obesity and by alterations in the regulation of leptin [36].

In addition to this, it was identified that the classification of people with non-infectious chronic diseases in the high-risk group for severe or even fatal COVID-19 is detrimental to their mental health; people with diabetes and/or symptoms of cardiovascular disease presented higher levels of depression (71.6%) and stress (73.7%) during the pandemic, compared to the general population [37]. Meanwhile, sleep disturbances were generated worldwide during the COVID-19 pandemic [38,39]; it was even identified that self-perception about the risk of contracting the infection, together with the anxiety derived from the pandemic, increased the possibility of developing sleep disturbances [40]. Specifically for CD, it was reported that during the COVID-19 pandemic, Polish people with high blood pressure and dyslipidemia had significantly higher levels of insomnia than the general population [41].

Currently, the increasing deterioration in mental health caused during the COVID-19 pandemic in the general population is worrisome; therefore, it is necessary to analyze in detail the alterations on the mental health of the entire population, especially in vulnerable groups such as people with CD, as well as to elucidate how the pandemic could exacerbate the low adherence to the prevention programs and treatments of these pathologies. Therefore, as a first step to address this issue, the present study determined the psychological factors associated with CD in Colombian adults during the COVID-19 pandemic. This detailed exploration of some of the psychological effects on mental health can be used to screen risks in this vulnerable sector of the population, as well as to generate programs for the prevention of disease and the promotion of health and well-being in times of a pandemic.

## 2. Materials and Methods

### 2.1. Study Design and Participants

An analytical and cross-sectional study was conducted, in which 284 people participated. The study was carried out between February 2021 and March 2021 and was approved by the Research Ethics Committee of the Antonio José Institute Camacho University Institution (FEV-001-21-01). Recruitment was carried out through the occupational health and safety area, approaching university personnel from different universities in Cali, Colombia. All participants received a detailed study information video via e-mail and provided written informed consent to participate in this study, which was conducted in accordance with the Declaration of Helsinki, good practices, and all applicable laws and regulations.

For the group with CD, 142 people who met the following inclusion criteria were included in the study: age ≥ 18 years; has some type of cardiovascular disease (heart attack, stroke, cerebral ischemia, cerebral infarction, cerebral thrombosis, cardiac arrhythmia, atrial fibrillation, heart failure, heart valve problems, arterial hypertension, etc.); hematological disease (purpura, hemophilia, etc.); and/or metabolic diseases (diabetes, dyslipidemia, obesity, etc.).

In relation to the group without CD, there were 142 people who met the following inclusion criteria: age ≥ 18 years; not having any type of cardiovascular, hematological or metabolic disease.

The exclusion criterion for both groups was: having other diseases such as asthma, COPD, cancer, chronic kidney disease, orphan or rheumatological diseases.

### 2.2. Outcome Variables

All the variables in this study were measured through an online form due to the current global situation.

#### 2.2.1. Quality of Life Related to Health

Health-related quality of life was measured using the SF-12v2 questionnaire that was validated in the Colombian population [42]. It consists of a total of 12 items that determine the state of physical and mental health in a positive or negative way through eight scales or domains: (i) physical role, (ii) physical functioning, (iii) general health, (iv) bodily pain, (v) social functioning, (vi) vitality, (vii) mental health and (viii) emotional role. Likewise, two summary scores were obtained, both physical and mental. The total score of this questionnaire ranges from 0 to 100, with the highest scores being a better health-related quality of life. In order to group those evaluated into those with a high level and those with a low level of health-related quality of life, the Colombian normative values were taken as a reference, so that two categories were established: scores < 50 as “by below average (BA)” and with scores ≥ 50 were classified as “above average (AA)”. Values above or below the mean were interpreted as better or worse health status, respectively, than the reference population [42].

#### 2.2.2. Sleep Quality

The Pittsburgh Sleep Quality Index (PSQI) [43] is a questionnaire that was used to assess sleep quality and was validated for the Colombian population [44]. This questionnaire consists of a total of 24 questions made up of 19 self-assessed questions and 5 that are answered by a roommate or housemate. In addition, 7 subscales or domains can be generated: (i) sleep latency, (ii) subjective sleep quality, (iii) dysfunctions during the day, (iv) sleep duration, (v) sleep disturbances, (vi) habitual sleep efficiency and (vii) use of sleep medications and a total score. Each of the questions in this questionnaire has a scale that ranges from 0 to 3, where poorer sleep quality is indicated by higher scores and the total score ranges from 0 to 21, with poor sleep quality being scores > 5 [43,44,45]. Taking into account that other studies used a cut-off score of 5 points, in the global PSQI score, to categorize people with or without sleep disorders [35,46]. In this research, for both groups, those evaluated were classified according to whether or not they presented with low sleeping quality (LSQ) in such a way that people with scores < 5 were categorized as without LSQ, while those with scores ≥ 5 were grouped in the with LSQ category.

#### 2.2.3. Depression

The Zung Self-Assessment Depression Scale (ZSDS) [47] was used to assess depression and was valid in Colombian populations [48]. This scale is made up of a self-administered survey with a total of 20 questions, of which 10 are negative and 10 are positive; each one refers to the frequency of depressive symptoms during the last two weeks and is rated on a scale of 1 to 4. Likewise, this scale indicates the four characteristics of depression, such as psychomotor activities, the dominant effect, the physiological equivalents and other alterations. The total score ranges between 20 and 80 points, with the highest scores indicating worse depression. Participants who obtained a score of 50 or more points were categorized as having depressive symptoms (DS). On the other hand, those who obtained less than the said score were categorized as without DS.

### 2.3. Covariates

#### 2.3.1. Sociodemographic and Anthropometric Data

A battery of questions was carried out on the demographic data of the participants: sex, age, marital status, urban or rural area (<150 inhabitants per km^2^) of residence, socioeconomic status [49], alcohol consumption and smoking, weight and height. With these last two data, the Body Mass Index (BMI) was obtained [50]. To assess the impact of the current COVID-19 pandemic on people with CD, variables related to exposure to the disease or people who have the disease were analyzed. In addition, each participant was asked if their housemates or family members are at risk of contracting COVID-19 or are at risk of developing severe or fatal COVID-19.

#### 2.3.2. Level of Physical Activity

The International Physical Activity Questionnaire (IPAQ) was used in its short version, which is used to measure the Physical Activity (PA) performed during the last 7 days and was validated and translated for the Colombian population [51]. This questionnaire reports the time in which an individual carries out a series of activities characterized by being vigorous and moderate. In addition, it evaluates three qualities of PA, such as the frequency, which refers to the days per week; the intensity, which can be mild, moderate or vigorous; and the duration, which refers to the time spent per day. The PA performed weekly is recorded in Mets per minute and week; its reference values are walking (3.3 Mets), moderate PA (4 Mets), and vigorous PA (8 Mets). In order to obtain the number of Mets, each of the aforementioned values (3.3, 4 or 8 Mets) must be multiplied by the time in minutes of the activity that is carried out in a day and by the number of days a week that it is carried out [52]. The results obtained in this questionnaire can be summarized in three categories: (i) high, which refers both to the performance of vigorous PA for 3 days or more with an energy expenditure of 1500 Mets × min/week and the performance of moderate PA with an expenditure energy of at least 3000 Mets × min/week; moderate, which can be defined on three occasions: moderate activity with an energy expenditure of at least 600 Mets × min/week for 5 days, moderate activity for 5 days with a duration of at least 30 min per day or vigorous PA with a duration of at least 25 min a day for 3 days or more; and finally, low, which occurs when neither of the two previous categories is met, or the participant does not perform PA. Taking into account this classification, the variable that refers to active (those classified with high and moderate levels) or insufficiently active (with a low level) was recategorized.

### 2.4. Sample Size Calculation

To calculate the sample size, at least 10 participants per independent variable were necessary for the logistic regression model [53]. In the present study, we considered 3 independent variables (health-related quality of life, sleep quality and depression), as well as 20 possible predictor variables (CD disease, sex, 7 BMI categories, 4 marital status categories, residence, 3 socioeconomic status categories, smoker, alcohol, living with people at risk of COVID-19 and the presence of COVID-19). Therefore, over 230 subjects were required for the purposes of our analysis. The final number of participants was 284.

### 2.5. Data Analysis

An exploratory analysis was carried out to identify the distribution of the data from the Kolmogorov–Smirnov test and the identification of extreme values. From the univariate analysis, the study population is characterized by presenting the independent and dependent variables in each group: control and patients with risk comorbidities for COVID-19. For continuous quantitative variables with normal distribution, the mean value (m) and its standard deviation (SD) are calculated, while for those that do not present a normal distribution, the median (me) and interquartile range (IQR) are calculated. The qualitative variables are presented with absolute numbers and percentages in each category. We proceeded to identify whether the independent variables between groups are comparable. Because they are independent groups, statistical methods such as chi-square or Fisher’s tests were used for categorical variables, as appropriate; for quantitative variables, evaluation was carried out with *t*-test (normal variables) or rank sum (non-normal variables) tests. Likewise, it was analyzed whether there is a relationship between the independent and dependent variables in the group of patients, as well as in the group of healthy ones. A simple model was performed to evaluate the association between having a CD and having sleep disturbances, depression and quality of life. The associated variables in the bivariate were integrated into multiple models, which were developed independently for each outcome variable and for each group (without CD and with CD). Finally, the most parsimonious model is presented that explains the association for each variable (depression and sleep quality alteration).

For all the statistical tests of hypothesis contrast, a significance level of 0.05 and a confidence level of 95% (95% CI) were established and were analyzed using the Stata 12.0 statistical package.

## 3. Results

**Figure 1:**
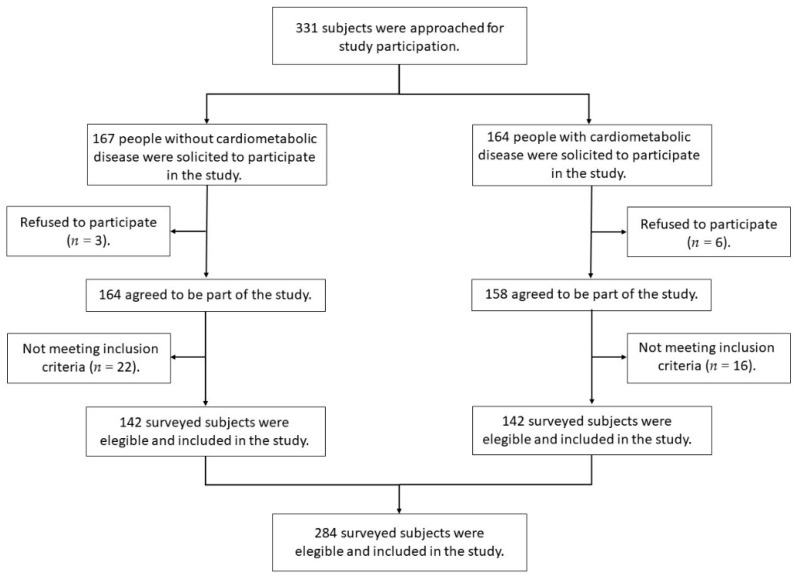
Flow diagram of study design.

A total of 332 subjects were contacted to participate in the study. Nine refused to participate in the study, and 39 did not meet the inclusion criteria. This left 284 participants who were divided into two groups, the first for people with cardiometabolic disease and the second for people without cardiometabolic diseases (Figure 1).

Table 1 shows the descriptive data of the population. In total, the sample was made up of 284 participants (64.75% men) who were grouped according to the presence or absence of CD into two balanced groups in terms of the number of participants. The categorical analysis in relation to the IBM showed that in the group without CD, 39.43% (*n* = 56) presented pre-obesity, while none of the respondents presented obesity 1, 2 or 3. In contrast, in the group with CD, 41.54% (*n* = 59) presented pre-obesity, 30.28% (*n* = 43) obesity 1, 9.85% (*n* = 14) obesity 2 and 1.40% (*n* = 2) obesity 3. In addition, regardless of the group without CD and with CD, the majority of those evaluated resided in urban areas and mostly belonged to the middle and high socioeconomic strata of the population; likewise, they lived with people at risk of developing serious or fatal COVID-19, were non-smokers and used alcohol occasionally. On the other hand, according to the IPAQ score, there was a higher proportion of insufficiently active people in the group without CD compared to the group with CD. A comparative analysis was carried out to verify that the sociodemographic variables between both groups do not present significant differences (*p* > 0.05) and thus allow comparability between independent groups. In order to determine the *p*-value for quantitative variables, a *t*-test was used, and a chi-square test for qualitative variables.

According to Table 2, the analysis of the mean differences between the people classified as with and without CD shows significant differences in quality of life, specifically related to the “physical health” dimension of the SF12v2. This is evidenced in most of the domains of this dimension (*p* < 0.05), with the exception of the “physical role” domain (*p* = 0.462). Likewise, differences were found in terms of sleep quality, notable in almost all the domains of the PSQI (*p* < 0.05), with the exception of the “sleep efficiency domain” (*p* = 0.276). In turn, it was shown that people with CD present significantly higher DS compared to the population without CD (*p* < 0.001). The preceding provides evidence of the association of said variables with the presence or absence of CD in the population evaluated.

The simple logistic regression model shown in Table 3 indicates that the main outcome variables associated with the CD population are the presence of LSQ and DS; according to the model, the chance of suffering from LSQ or DS is 3.51 or 1.98 times greater in people with CD, compared to people without CD.

The bivariate analysis showed that the variables that presented an association (*p* < 0.05) with depressive symptoms (Zung) in persons with cardiometabolic diseases were BMI and IPAQ, while for the group without cardiometabolic disease, the association was only observed with sex and living with people at risk of COVID-19. In people without CD, the PSQI was associated with alcohol consumption frequency. For those with CD, PSQI was associated with BMI and living with people at risk of COVID-19.

For each simple model, all the variables were adjusted for age, finding that regardless of age, the association is preserved. These variables that showed association were applied to the multiple models.

Table 4 shows the two final parsimonious multiple logistic regression models, which explain the association of the most significant variables, both for the presence of DS and for the presence of LSQ, in the two populations studied: with and without CD. According to these models, it is important to point out that for DS-ZSDS, there are no common variables that explain the presence of DS in the two populations. For the population without CD, the opportunity to present DS for men is 3.21 times greater than for women and 4.86 times greater when living with people at risk of developing serious or fatal COVID-19. On the other hand, in the population with CD, the opportunity to present DS in people with a high BMI is 2.45 times greater compared to people who present a low BMI.

According to the model for LSQ-PSQI, the variable “Living with people at risk of COVID-19” fully explains the presence of LSQ in the population without CD, in whom the probability of presenting with LSQ is 4.22 times greater than that in those who do not live with people at risk for severe COVID-19. Similarly, in the population with CD, the opportunity to develop LSQ is 3.64 times more due to the previously mentioned variable; additionally, the opportunity to present LSQ in people with a high BMI is 5.88 times greater compared to people who present a low BMI.

In the population without CD, living with people at risk of developing severe or fatal COVID-19 affects the presence of LSQ to a greater extent compared to DS (OR = 4.86 vs. OR = 4.22, respectively). On the other hand, in the population with CD, a high BMI affects the presence of LSQ more noticeably compared to DS (OR = 5.88 vs. OR = 2.45, respectively).

## 4. Discussion

Cardiometabolic Diseases (CD) are among the leading causes of death and disability in the world, where people with CD represent a particularly vulnerable population as they are at increased risk of developing depression or anxiety [54,55]. International reports state that during the COVID-19 pandemic, the prognosis to develop severe forms of this disease is strongly related to having cardiometabolic disorders [56]. Furthermore, given that generalized outbreaks of infectious diseases have psychological implications [57], the importance of the association between psychological disorders and CD has aroused greater interest.

Thus, in this study, the psychological factors associated with CD on Colombian adults during the COVID-19 pandemic were determined. The main contributions of this work confirmed that during the COVID-19 pandemic situation, the population with CD had a greater probability of presenting DS, as well as an LSQ compared to the population without CD; likewise, BMI was identified as an important variable to present an LSQ and DS in people with CD. Together, these findings can be used to generate health and well-being monitoring, prevention and promotion programs that tend towards an optimal approach to mental health in times of a pandemic.

It was described that during pandemics, the self-perceived susceptibility and severity of presenting severe disease, as well as greater confidence in the effectiveness of the recommended measures against the disease, are determinants for adherence to preventive behaviors, such as performance of PA [58,59,60,61]. This could explain the findings related to PA levels, where a higher proportion of people with CD were active (64.79%) compared to people without CD (47.89%); it is possible that people with CD increased or maintained their PA levels during the pandemic, knowing that PA is associated with reduced cardiovascular risk and improved cardiometabolic risk factors [62], and that physical inactivity is an important modifiable risk factor associated with more severe clinical conditions for COVID-19 [63].

Likewise, it was found that 41.53% of the population with CD presented a BMI ≥ 30 despite the fact that 64.79% of the people with CD were physically active. These findings can be explained by changes in body composition that do not depend solely on the PA level but on other factors such as eating habits and genetics [64].

On the other hand, it was proposed that the presence of DS in people with CD may be influenced by various factors; among these, behavioral aspects such as inactive lifestyle and poor eating habits as well as environmental and cultural risk factors were identified [65]. In the literature, it was established that people with thyroid disease, diabetes, cardiovascular disease, and other chronic medical conditions have a higher risk of presenting depressive disorders [54,55,66,67]; in the same way, depression is present in one of every four people with type 2 diabetes mellitus [68]. The foregoing supports the findings presented in which the chance of suffering from DS when presenting with CD was 1.98 times, compared with not presenting CD.

Additionally, it was suggested that potential risk factors for depression in people with CD often interact with each other [27]; for example, obesity and depression often coexist, increasing the risk of cardiovascular disease [69]. This coincides with the results of this study, in which it was identified that the probability of presenting DS is higher for those with high BMI.

In addition, the sex variable was related to the presence of DS in the population without CD, with the likelihood of DS being 3.21 times greater for men than for women. These findings differ from those found in several studies, where depressive symptoms were found to be greater in women than in men [5,8,41,70]. Because of this, it is of great importance that mental health programs include risk assessments and screening for depression, as well as the management of personalized approaches for male depression [71]. More research is needed to elucidate the causal mechanisms underlying these associations.

In recent years, interest has grown around the possible link between cardiovascular diseases, type 2 diabetes mellitus and obesity with LSQ [72], different factors that contribute to LSQ and psychological problems. One of the most important risk factors for LSQ during the COVID-19 pandemic is having an underlying disease, such as CD; a study carried out in Poland found significant differences in relation to insomnia between people with and without hypertension (*p* = 0.006) and between those with and without dyslipidemia (*p* = 0.035) during this pandemic [41]. This agrees with our results in which significant differences were found in relation to LSQ between those with and without CD (*p* < 0.001); the opportunity to suffer from LSQ when presenting with CD is 3.51 times more compared to not presenting with CD.

On the other hand, the variable “living with people at risk of COVID-19” was associated with suffering from LSQ and DS. Regardless of the presence or not of CD, living with a person with comorbidities for COVID-19 represents in itself a constant concern that threatens the mental health of the population, which is why local and regional organizations address these psychosocial challenges holistically [5].

Similarly, obesity can bring with it diseases that deteriorate the quality of sleep, such as obstructive sleep apnea. Obesity and respiratory sleep disorders can increase the morbidity and severity of complications, such as hypertension and type 2 diabetes mellitus [73]; this could explain our results in which it was identified that the probability of presenting DS and LSQ is higher for those with high BMI.

Finally, we identified some limitations of the study in the sense that factors related to the pandemic were not examined that could help to give greater clarity to the statistical models obtained; for example, assessing anxiety and the influence of risk self-perception of severe or fatal COVID-19 in people with CD or including data related with the comorbid conditions of the patients. On the other hand, the data analyzed in this investigation were obtained from self-completed surveys, so the influence of recall bias cannot be ruled out [74]. Finally, as it is a cross-sectional study, it does not allow us to assess whether there was any change in terms of mental health from before the pandemic to the current date.

## 5. Conclusions

This study showed that people with a CD diagnosis were more physically active during the COVID-19 pandemic than people without CD. Likewise, it was shown that people with CD are more likely to have LSQ and DS than people without CD. Furthermore, in this same population, the probability of presenting DS and LSQ is higher for those with high BMI. Finally, living with people at risk of COVID-19 increases the probability of presenting LSQ both for the population with CD and for those without CD.

Thus, a comprehensive and complete approach to CD patients is suggested during this pandemic, in which it does not focus on the underlying pathologies but also on the importance of mental health. The findings of this research may be used to generate programs for monitoring, disease prevention and the promotion of health and well-being, which allow a correct approach to mental health in times of a pandemic.

## Figures and Tables

**Table 1:** Descriptive data of the sample (*n* = 284).

				Without CD (*n* = 142)	With CD (*n* = 142)	*p*
Age. mean (SD)				41.53 (10.06)	48.15 (10.15)	1.000
Sex. *n* (%)	Male			74 (52.11)	84 (59.15)	0.232
	Female			68 (47.89)	58 (40.85)	
* BMI. *n* (%)	Underweight			1 (0.07)	1 (0.07)	0.000
	Normal range			84 (59.15)	15 (10.56)	
	Overweight			1 (0.07)	8 (5.63)	
	Preobese			56 (39.43)	59 (41.54)	
	Obese class 1			0 (0)	43 (30.28)	
	Obese class 2			0 (0)	14 (9.85)	
	Obese class 3			0 (0)	2 (1.40)	
Marital status. *n* (%)	Single/Free union			84 (59.16)	64 (45.97)	0.113
	Married			46 (32.39)	66 (46.48)	
	Separated/widower			12 (8.45)	12 (8.45)	
Residence. *n* (%)	Rural area			18 (12.68)	19 (13.38)	
	Urban area			124 (87.32)	123 (86.62)	
** Socioeco-nomic strata *n* (%)	Low			6 (4.23)	15 (9.15)	0.069
	Medium			76 (53.52)	77 (54.22)	
	High			60 (42.25)	50 (35.21)	
Smoker. *n* (%)	No			138 (97.18)	141 (99.30)	0.175
	Yes			4 (2.82)	1 (0.70)	
Alcohol. *n* (%)	Not consumption			60 (42.25)	67 (47.18)	0.501
	Yes consumption			82 (57.74)	75 (52.82)	
		Frequency	Occasional	77 (54.23)	69 (48.59)	
			Frequently	5 (3.51)	6 (4.23)	
Living with people at risk of COVID-19. *n* (%)	No			97 (68.31)	96 (67.61)	0.898
	Yes			45 (31.69)	46 (32.39)	
Presence of COVID-19 symptoms. *n* (%)	No			142 (100.00)	141 (99.30)	0.316
	Yes			0 (0.00)	1 (0.70)	
IPAQ. *n* (%)	Active			68 (47.89)	92 (64.79)	0.004
	Insufficiently active			74 (52.11)	50 (35.21)	

CD: Cardiometabolic diseases. BMI (kg/m^2^): body mass index. IPAQ: The International Physical Activity Questionnaire. * According to the WHO classification. ** According to Law 142 of 1994 that establishes the Regime of Domiciliary Public Services in Colombia. SD: standard deviation.

**Table 2:** Comparison of outcome variables according to cardiometabolic disease classification (without CD and with CD).

			Without CD (*n* = 142)	With CD (*n* = 142)	*p*
SF-12v2. mean (SD)	Physical Health dimension	Physical functioning	94.36 (13.44)	82.21 (26.39)	0.000
		Physical role	87.32 (31.19)	84.50 (33.25)	0.462
		Bodily pain	91.90 (15.62)	87.85 (17.79)	0.042
		General health	81.52 (14.65)	63.55 (21.61)	0.000
		PCS	88.77 (13.40)	79.53 (16.82)	0.000
	Mental Health dimension	Vitality	77.04 (19.19)	76.05 (21.20)	0.681
		Social functioning	89.08 (17.47)	86.26 (22.98)	0.246
		Emotional role	90.14 (28.08)	84.85 (31.50)	0.137
		Mental health	77.60 (17.86)	75.84 (20.42)	0.440
		MCS	83.46 (15.21)	80.75 (18.23)	0.174
	Total score for SF-12v2		86.53 (12.88)	81.03 (16.56)	0.002
SF-12v2. *n* (%)	Quality of life BA		3 (2.11)	9 (6.34)	0.076
	Quality of life AA		139 (97.89)	133 (93.66)	0.076
PSQI. mean (SD)	Sleep quality domain		0.58 (0.66)	1.09 (0.68)	0.000
	Sleep latency domain		0.52 (0.69)	1.38 (0.85)	0.000
	Sleep duration domain		0.76 (0.69)	1.50 (0.81)	0.000
	Sleep efficiency domain		0.48 (0.81)	0.58 (0.70)	0.276
	Sleep disturbances domain		0.66 (0.63)	1.17 (0.67)	0.000
	Use of sleeping medication domain		0.35 (0.66)	0.56 (0.64)	0.009
	Daytime dysfunction domain		0.30 (0.47)	0.73 (0.73)	0.000
	Total score for PSQI		3.69 (3.41)	7.00 (3.34)	0.000
PSQI. *n* (%)	Without LSQ		97 (68.31)	54 (38.03)	0.000
	With LSQ		45 (31.69)	88 (61.97)	0.000
ZSDS. *n* (%)	Without DS		89 (62.68)	65 (45.77)	0.000
	With DS		53 (37.32)	77 (54.23)	0.000

CD: cardiometabolic diseases. DS: depressive symptoms. LSQ: low sleeping quality. SF-12v2: 12-Item Short-Form Health Survey. PCS: physical health component summary. MCS: mental health component summary. BA: below average. AA: above average. PSQI: Pittsburgh Sleep Quality index. ZSDS: Zung Self-Rating Depression Scale. SD: standard deviation.

**Table 3:** Association between people with CD and outcome variables (BA -SF-12v2; LSQ-PSQI and DS-ZSDS).

	OR	*p* > |z|	[95% CI] Min–Max
BA-SF-12v2	0.32	0.09	0.08–1.20
LSQ-PSQI	3.51	0.00	2.15–5.73
DS-ZSDS	1.98	0.00	1.24–3.19

CD: cardiometabolic diseases. DS: depressive symptoms. LSQ: low sleeping quality. BA: below average. SF-12v2: 12-Item Short-Form Health Survey. PSQI: Pittsburgh Sleep Quality index. ZSDS: Zung Self-Rating Depression. OR: odds ratio. CI: confidence interval. min: minimum. max: maximum.

**Table 4:** Final multiple logistic regression model for DS (ZSDS) and LSQ (PSQI) in people with and without CD.

		Without CD			With CD		
		OR	[95% CI]	*p* > |z|	OR	[95% CI]	*p* > |z|
			min–max			min–max	
DS-ZSDS	Sex (male)	3.21	1.45–7.12	0.004	—	—	—
	Living with people at risk of COVID-19 (Yes)	4.86	2.14–11.03	0.000	—	—	—
	* BMI	—	—	—	2.45	1.66–3.60	0.000
	Cons	0.18	0.09–0.37	0.000	0.02	0.01–0.12	0.000
LSQ-PSQI	Living with people at risk of COVID-19 (yes)	4.22	2.58–6.91	0.000	3.64	1.17–11.28	0.025
	* BMI	—	—	—	5.88	1.15–2.74	0.000
	Cons	0.01	0.00–0.07	0.000	0.00	0.00–0.01	0.000

CD: cardiometabolic diseases. DS: depressive symptoms. LSQ: low sleeping quality. ZSDS: Zung Self-Rating Depression Scale. PSQI: Pittsburgh Sleep Quality index. BMI (kg/m^2^): body mass index. * According to the WHO classification. (—): Does not apply. OR: odds ratio. CI: confidence interval. min: minimum. max: maximum.

## References

[1] OPS/OMS Actualización Epidemiológica: Enfermedad por Coronavirus (COVID-19)—19 de Junio, 2021. https://www.paho.org/es/documentos/actualizacion-epidemiologica-enfermedad-por-coronavirus-covid-19-19-junio-2021.

[2] PAHO/WHO Epidemiological Update: Variants of SARS-CoV-2 in the Americas. 26 January 2021. https://iris.paho.org/bitstream/handle/10665.2/53239/EpiUpdate26January2021_eng.pdf?sequence=1&isAllowed=y.

[3] Pinilla-Roncancio M. (2018). The reality of disability: Multidimensional poverty of people with disability and their families in Latin America. Disabil. Health J..

[4] Holmes E.A., O’Connor R.C., Perry V.H., Tracey I., Wessely S., Arseneault L., Ballard C., Christensen H., Silver R.C., Everall I. (2020). Multidisciplinary research priorities for the COVID-19 pandemic: A call for action for mental health science. Lancet Psychiatry.

[5] Hossain M.M., Tasnim S., Sultana A., Faizah F., Mazumder H., Zou L., McKyer E.L.J., Ahmed H.U., Ma P. (2020). Epidemiology of mental health problems in COVID-19: A review. F1000Research.

[6] Torales J., O’Higgins M., Castaldelli-Maia J.M., Ventriglio A. (2020). The outbreak of COVID-19 coronavirus and its impact on global mental health. Int. J. Soc. Psychiatry.

[7] Grolli R.E., Mingoti M.E.D., Bertollo A.G., Luzardo A.R., Quevedo J., Réus G.Z., Ignácio Z.M. (2021). Impact of COVID-19 in the Mental Health in Elderly: Psychological and Biological Updates. Mol. Neurobiol..

[8] Klimkiewicz A., Schmalenberg A., Klimkiewicz J., Jasińska A., Jasionowska J., Machura W., Wojnar M. (2021). COVID-19 Pandemic Influence on Healthcare Professionals. J. Clin. Med..

[9] Singh S., Roy D., Sinha K., Parveen S., Sharma G., Joshi G. (2020). Impact of COVID-19 and lockdown on mental health of children and adolescents: A narrative review with recommendations. Psychiatry Res..

[10] Zhou S.J., Zhang L.G., Wang L.L., Guo Z.C., Wang J.Q., Chen J.C., Liu M., Chen X., Chen J.X. (2020). Prevalence and socio-demographic correlates of psychological health problems in Chinese adolescents during the outbreak of COVID-19. Eur. Child Adolesc. Psychiatry.

[11] Wu T., Jia X., Shi H., Niu J., Yin X., Xie J., Wang X. (2021). Prevalence of mental health problems during the COVID-19 pandemic: A systematic review and meta-analysis. J. Affect. Disord..

[12] Rangaraj V.R., Knutson K.L. (2016). Association between sleep deficiency and cardiometabolic disease: Implications for health disparities. Sleep Med..

[13] WHO (2020). Noncommunicable Diseases: Progress Monitor 2020.

[14] Zurique Sánchez M.S., Zurique Sánchez C.P., Camacho López P.A., Sanchez Sanabria M., Hernández Hernández S.C. (2019). Prevalencia de hipertensión arterial en Colombia: Revisión sistemática y meta-análisis. Acta Méd. Colomb..

[15] Minsalud. Obesidad, un Factor de Riesgo en el COVID-19. https://www.minsalud.gov.co/Paginas/Obesidad-un-factor-de-riesgo-en-el-covid-19.aspx.

[16] Gómez L.F., Mora M., Riascos S., Parra D. (2019). Prevalencias de diabetes e hipertensión en Colombia: Un revisión sistemática. Rev. Fac. Nac. De Salud Pública.

[17] Pascual-Sánchez A., Caballo-Escribano C. (2017). Funcionamiento y calidad de vida en personas con enfermedades crónicas: Poder predictivo de distintas variables psicológicas. J. Enfermería Glob..

[18] Damares-Garcia G., Alcalá-Pompeo D., Palota-Eid L., Bernardi-Cesarino C., Pinto M.H., Gonçalves L.W.P. (2018). Relationship between anxiety, depressive symptoms and compulsive overeating disorder in patients with cardiovascular diseases. Rev. Lat. Am. De Enferm..

[19] Choi Y., Choi J.W. (2020). Association of sleep disturbance with risk of cardiovascular disease and all-cause mortality in patients with new-onset type 2 diabetes: Data from the Korean NHIS-HEALS. Cardiovasc. Diabetol..

[20] Cappuccio F.P., Miller M.A. (2017). Sleep and Cardio-Metabolic Disease. Curr. Cardiol. Rep..

[21] Khaledi M., Haghighatdoost F., Feizi A., Aminorroaya A. (2019). The prevalence of comorbid depression in patients with type 2 diabetes: An updated systematic review and meta-analysis on huge number of observational studies. Acta Diabetol..

[22] Ho A.K., Thorpe C.T., Pandhi N., Palta M., Smith M.A., Johnson H.M. (2015). Association of anxiety and depression with hypertension control: A US multidisciplinary group practice observational study. J. Hypertens..

[23] Li Z., Li Y., Chen L., Chen P., Hu Y. (2015). Prevalence of Depression in Patients With Hypertension: A Systematic Review and Meta-Analysis. Medicine.

[24] Mejia-Lancheros C., Estruch R., Martínez-González M.A., Salas-Salvadó J., Corella D., Gómez-Gracia E., Fiol M., Santos J.M., Fitó M., Arós F. (2014). Blood pressure values and depression in hypertensive individuals at high cardiovascular risk. BMC Cardiovasc. Disord..

[25] Endomba F.T., Mazou T.N., Bigna J.J. (2020). Epidemiology of depressive disorders in people living with hypertension in Africa: A systematic review and meta-analysis. BMJ Open.

[26] Lloyd C.E., Roy T., Nouwen A., Chauhan A.M. (2012). Epidemiology of depression in diabetes: International and cross-cultural issues. J. Affect. Disord..

[27] Mukeshimana M., McHunu G. (2017). Management of Co-Morbidity of Depression and Chronic Non-Communicable Diseases in Rwanda. Ethiop. J. Health Sci..

[28] Wright J., Mazumdar P., Barua D., Lina S., Bibi H., Kanwal A., Mujeeb F., Naz Q., Safi R., Ul Haq B. (2020). Integrating depression care within NCD provision in Bangladesh and Pakistan: A qualitative study. Int. J. Ment. Health Syst..

[29] Eze-Nliam C.M., Thombs B.D., Lima B.B., Smith C.G., Ziegelstein R.C. (2010). The association of depression with adherence to antihypertensive medications: A systematic review. J. Hypertens..

[30] Wang P.S., Bohn R.L., Knight E., Glynn R.J., Mogun H., Avorn J. (2002). Noncompliance with antihypertensive medications: The impact of depressive symptoms and psychosocial factors. J. Gen. Intern. Med..

[31] Bauer A.M., Parker M.M., Moffet H.H., Schillinger D., Adler N.E., Adams A.S., Schmittdiel J.A., Katon W.J., Karter A.J. (2017). Depressive symptoms and adherence to cardiometabolic therapies across phases of treatment among adults with diabetes: The Diabetes Study of Northern California (DISTANCE). Patient Prefer. Adherence.

[32] WHO (2019). Integrating the Prevention, Treatment and Care of Mental Health Conditions and Other Noncommunicable Diseases within Health Systems. https://www.euro.who.int/__data/assets/pdf_file/0004/397786/Mental-Health-Conditions-ENG.pdf.

[33] Brocato J., Wu F., Chen Y., Shamy M., Alghamdi M.A., Khoder M.I., Alkhatim A.A., Abdou M.H., Costa M. (2015). Association between sleeping hours and cardiometabolic risk factors for metabolic syndrome in a Saudi Arabian population. BMJ Open.

[34] Lee S.W.H., Ng K.Y., Chin W.K. (2017). The impact of sleep amount and sleep quality on glycemic control in type 2 diabetes: A systematic review and meta-analysis. Sleep Med. Rev..

[35] Rusu A., Ciobanu D., Bala C., Cerghizan A., Roman G. (2019). Social jetlag, sleep-related parameters, and glycemic control in adults with type 1 diabetes: Results of a cross-sectional study. J. Diabetes.

[36] Mosavat M., Mirsanjari M., Arabiat D., Smyth A., Whitehead L. (2021). The Role of Sleep Curtailment on Leptin Levels in Obesity and Diabetes Mellitus. Obes. Facts.

[37] Sayeed A., Kundu S., Al Banna M.H., Christopher E., Hasan M.T., Begum M.R., Chowdhury S., Khan M.S.I. (2020). Mental Health Outcomes of Adults with Comorbidity and Chronic Diseases during the COVID-19 Pandemic: A Matched Case-Control Study. Psychiatr. Danub..

[38] Medina-Ortiz O., Araque-Castellanos F., Ruiz-Domínguez L.C., Riaño-Garzón M., Bermudez V. (2020). Trastornos del sueño a consecuencia de la pandemia por COVID-19. J Rev. Peru. De Med. Exp. Y Salud Publica.

[39] Mandelkorn U., Genzer S., Choshen-Hillel S., Reiter J., Meira E.C.M., Hochner H., Kheirandish-Gozal L., Gozal D., Gileles-Hillel A. (2021). Escalation of sleep disturbances amid the COVID-19 pandemic: A cross-sectional international study. J. Clin. Sleep Med. JCSM Off. Publ. Am. Acad. Sleep Med..

[40] Ara T., Rahman M.M., Hossain M.A., Ahmed A. (2020). Identifying the Associated Risk Factors of Sleep Disturbance During the COVID-19 Lockdown in Bangladesh: A Web-Based Survey. Front. Psychiatry.

[41] Wańkowicz P., Szylińska A., Rotter I. (2021). The Impact of the COVID-19 Pandemic on Psychological Health and Insomnia among People with Chronic Diseases. J. Clin. Med..

[42] Ramírez-Vélez R., Agredo-Zuñiga R.A., Jerez-Valderrama A.M. (2010). The reliability of preliminary normative values from the short form health survey (SF-12) questionnaire regarding Colombian adults. Rev. De Salud Publica.

[43] Buysse D.J., Reynolds C.F., Monk T.H., Berman S.R., Kupfer D.J. (1989). The Pittsburgh Sleep Quality Index: A new instrument for psychiatric practice and research. Psychiatry Res..

[44] Escobar-Córdoba F., Eslava-Schmalbach J. (2005). Colombian validation of the Pittsburgh Sleep Quality Index. Rev. De Neurol..

[45] Doi Y., Minowa M., Uchiyama M., Okawa M., Kim K., Shibui K., Kamei Y. (2000). Psychometric assessment of subjective sleep quality using the Japanese version of the Pittsburgh Sleep Quality Index (PSQI-J) in psychiatric disordered and control subjects. Psychiatry Res..

[46] Çalışkan F., Toker İ., Tur B., Hacar S., Türe B. (2015). Assessment of the Pittsburgh Sleep Quality Index among Physician’s Speciality Who Work Night Shifts. Emerg. Med. Open J..

[47] Zung W.W. (1965). A self-rating depression scale. Arch. Gen. Psychiatry.

[48] Campo-Arias A., Diaz Martinez L., Rueda-Jaimes G., Barros J. (2005). Validación de la escala de Zung para depresión en universitarias de Bucaramanga, Colombia. Rev. Colomb. Psiquiatr..

[49] Colombia G.d. Ley 142 de 1994. https://www.funcionpublica.gov.co/eva/gestornormativo/norma.php?i=2752.

[50] WHO 10 Datos Sobre La Obesidad. https://www.who.int/features/factfiles/obesity/facts/es/.

[51] Craig C.L., Marshall A.L., Sjöström M., Bauman A.E., Booth M.L., Ainsworth B.E., Pratt M., Ekelund U., Yngve A., Sallis J.F. (2003). International physical activity questionnaire: 12-country reliability and validity. Med. Sci. Sports Exerc..

[52] Fan M., Lyu J., He P. (2014). Guidelines for data processing and analysis of the International Physical Activity Questionnaire (IPAQ). 2005. Zhonghua Liu Xing Bing Xue Za Zhi Zhonghua Liuxingbingxue Zazhi.

[53] Calvo M.O., Domínguez A.C. (2002). Regresión logística no condicionada y tamaño de muestra: Una revisión bibliográfica. Rev. Esp. Salud Publica.

[54] Zhang Y., Chen Y., Ma L. (2018). Depression and cardiovascular disease in elderly: Current understanding. J. Clin. Neurosci. Off. J. Neurosurg. Soc. Australas..

[55] Shinn E.H., Poston W.S., Kimball K.T., St Jeor S.T., Foreyt J.P. (2001). Blood pressure and symptoms of depression and anxiety: A prospective study. Am. J. Hypertens..

[56] Tian W., Jiang W., Yao J., Nicholson C.J., Li R.H., Sigurslid H.H., Wooster L., Rotter J.I., Guo X., Malhotra R. (2020). Predictors of mortality in hospitalized COVID-19 patients: A systematic review and meta-analysis. J. Med. Virol..

[57] Rajkumar R.P. (2020). COVID-19 and mental health: A review of the existing literature. Asian J. Psychiatry.

[58] Bish A., Michie S. (2010). Demographic and attitudinal determinants of protective behaviours during a pandemic: A review. Br. J. Health Psychol..

[59] Li S., Feng B., Liao W., Pan W. (2020). Internet Use, Risk Awareness, and Demographic Characteristics Associated With Engagement in Preventive Behaviors and Testing: Cross-Sectional Survey on COVID-19 in the United States. J. Med. Internet Res..

[60] González-Castro J.L., Ubillos-Landa S., Puente-Martínez A., Gracia-Leiva M. (2021). Perceived Vulnerability and Severity Predict Adherence to COVID-19 Protection Measures: The Mediating Role of Instrumental Coping. Front. Psychol..

[61] Dwyer M.J., Pasini M., De Dominicis S., Righi E. (2020). Physical activity: Benefits and challenges during the COVID-19 pandemic. Scand. J. Med. Sci. Sports.

[62] Swift D.L., McGee J.E., Earnest C.P., Carlisle E., Nygard M., Johannsen N.M. (2018). The Effects of Exercise and Physical Activity on Weight Loss and Maintenance. Prog. Cardiovasc. Dis..

[63] Sallis R., Young D.R., Tartof S.Y., Sallis J.F., Sall J., Li Q., Smith G.N., Cohen D.A. (2021). Physical inactivity is associated with a higher risk for severe COVID-19 outcomes: A study in 48 440 adult patients. Br. J. Sports Med..

[64] Kriaucioniene V., Petkeviciene J., Raskiliene A. (2019). Nutrition and physical activity counselling by general practitioners in Lithuania, 2000–2014. BMC Fam. Pract..

[65] Sarris J., O’Neil A., Coulson C.E., Schweitzer I., Berk M. (2014). Lifestyle medicine for depression. BMC Psychiatry.

[66] Carney R.M., Freedland K.E., Veith R.C. (2005). Depression, the autonomic nervous system, and coronary heart disease. Psychosom. Med..

[67] Dolynna O.V. (2013). Autonomic nervous system unbalance in patients with ischemic heart disease and depression. Likars’ka Sprav..

[68] Semenkovich K., Brown M.E., Svrakic D.M., Lustman P.J. (2015). Depression in type 2 diabetes mellitus: Prevalence, impact, and treatment. Drugs.

[69] Faulconbridge L.F., Driscoll C.F.B., Hopkins C.M., Bailer Benforado B., Bishop-Gilyard C., Carvajal R., Berkowitz R.I., DeRubeis R., Wadden T.A. (2018). Combined Treatment for Obesity and Depression: A Pilot Study. Obesty.

[70] Lazarevich I., Irigoyen Camacho M.E., Velázquez-Alva M.C., Flores N.L., Nájera Medina O., Zepeda Zepeda M.A. (2018). Depression and food consumption in Mexican college students. Nutr. Hosp..

[71] Oliffe J.L., Rossnagel E., Seidler Z.E., Kealy D., Ogrodniczuk J.S., Rice S.M. (2019). Men’s Depression and Suicide. Curr. Psychiatry Rep..

[72] Knowlden A.P., Higginbotham J.C., Grandner M.A., Allegrante J.P. (2021). Modeling Risk Factors for Sleep- and Adiposity-Related Cardiometabolic Disease: Protocol for the Short Sleep Undermines Cardiometabolic Health (SLUMBRx) Observational Study. JMIR Res. Protoc..

[73] Meurling I.J., Shea D.O., Garvey J.F. (2019). Obesity and sleep: A growing concern. Curr. Opin. Pulm. Med..

[74] Raphael K. (1987). Recall Bias: A Proposal for Assessment and Control. Int. J. Epidemiol..

